# Effect of bihemispheric transcranial direct current stimulation on distal upper limb function and corticospinal tract excitability in a patient with subacute stroke: a case study

**DOI:** 10.3389/fresc.2023.1250579

**Published:** 2023-09-05

**Authors:** Takahiro Shiba, Naomichi Mizuta, Naruhito Hasui, Yohei Kominami, Tomoki Nakatani, Junji Taguchi, Shu Morioka

**Affiliations:** ^1^ Department of Therapy, Takarazuka Rehabilitation Hospital, Medical Corporation SHOWAKAI, Hyogo, Japan; ^2^ Department of Rehabilitation, Faculty of Health Sciences, Nihon Fukushi University, Aichi, Japan; ^3^ Neurorehabilitation Research Center, Kio University, Nara, Japan; ^4^ Department of Neurorehabilitation, Graduate School of Health Sciences, Kio University, Nara, Japan

**Keywords:** intermuscular coherence, fugl-Meyer assessment, subacute stroke, transcranial direct current stimulation, stroke rehabilitation

## Abstract

**Introduction:**

Activation of the unaffected hemisphere contributes to motor function recovery post stroke in patients with severe upper limb motor paralysis. Transcranial direct current stimulation (tDCS) has been used in stroke rehabilitation to increase the excitability of motor-related areas. tDCS has been reported to improve upper limb motor function; nonetheless, its effects on corticospinal tract excitability and muscle activity patterns during upper limb exercise remain unclear. Additionally, it is unclear whether simultaneously applied bihemispheric tDCS is more effective than anodal tDCS, which stimulates only one hemisphere. This study examined the effects of bihemispheric tDCS training on corticospinal tract excitability and muscle activity patterns during upper limb movements in a patient with subacute stroke.

**Methods:**

In this single-case retrospective study, the Fugl–Meyer Assessment, Box and Block Test, electromyography, and intermuscular coherence measurement were performed. Intermuscular coherence was calculated at 15–30 Hz, which reflects corticospinal tract excitability.

**Results:**

The results indicated that bihemispheric tDCS improved the Fugl–Meyer Assessment, Box and Block Test, co-contraction, and intermuscular coherence results, as compared with anodal tDCS. Discussion: These results reveal that upper limb training with bihemispheric tDCS improves corticospinal tract excitability and muscle activity patterns in patients with subacute stroke.

## 1. Introduction

Only 5%–20% of patients completely recover from post-stroke upper limb motor paralysis (1, 2), and 25%–74% of stroke survivors require assistance with activities of daily living (3). In upper limb motor paralysis, the distal areas are last to recover (4–6). Even after partial recovery, muscle activity patterns during distal movement remain abnormal (7). During reaching movements, abnormal movement patterns can also manifest in the distal body parts, in conjunction with proximal movements (8). The most representative marker of recovery from upper limb motor paralysis is projection of corticospinal tract (CST) excitability to the extensor muscles of the fingers (9, 10). The CST is mainly involved in motor control of the contralateral distal areas. Enhancing CST excitability originating from the injured hemisphere is essential for recovery from upper limb motor dysfunction in patients post stroke. However, after stroke, muscles are simultaneously abnormally activated, and increased co-contraction of the agonist and antagonist muscles is observed (11, 12). Such co-contraction of the elbow muscles increases during voluntary movements on the affected side, preventing independent muscle contraction (13). Particularly, the co-contraction rate of the agonist and antagonist muscles of the affected wrist may be increased (14). Recent studies suggest that an increased muscle co-contraction index correlates with an impaired CST and that the increased co-contraction may be of cortical origin (15). In other words, CST produces selective movement, and damage to the CST may increase the co-contraction index, resulting in loss of selective movement.

The interhemispheric mechanisms contributing to the post-stroke upper limb movement recovery vary, depending on motor paralysis severity (16). Normally, during unilateral upper limb movements, increased excitability of the contralateral hemisphere inhibits the excitability of the ipsilateral hemisphere; in contrast, post stroke, decreased inhibition from the injured to the contralateral side leads to an increased excitability of the contralateral hemisphere (17–19). Additionally, motor-related regional activity in the non-injured hemisphere decreases with recovery from motor paralysis, whilst the interhemispheric activity balance improves (20, 21). Furthermore, excitation of the non-injured hemisphere has been reported to positively correlate with the Fugl–Meyer Assessment (FMA) score (22). Therefore, the activity in the motor-related areas of both the damaged and undamaged cerebral hemispheres may influence CST and selective movements.

Recently, transcranial direct current stimulation (tDCS), a non-invasive brain stimulation technique, has been reported to increase motor-related area activity in the injured hemisphere in patients post stroke (23–25). tDCS, a non-invasive technique, involves application of a weak direct current over the scalp to modulate cortical excitability. Anodal tDCS of the primary motor cortex (M1) in the injured hemisphere can increase motor evoked potentials (MEPs) and selective movement of the main motor muscles (7, 26, 27), whereas cathodal tDCS suppresses MEPs (28, 29). Anodal tDCS in combination with upper limb motor training reportedly leads to an increased improvement in upper limb motor function and cortical activity, compared to upper limb motor training alone (30). The increase in MEPs due to the application of anodal tDCS to the motor cortex is paralleled by an increase in intermuscular coherence in the beta frequency band (31). Furthermore, intermuscular coherence in the beta band reportedly reflects CST excitability and is highly correlated with MEPs (12). Therefore, we believe that intermuscular coherence analysis can detect changes in motor cortex excitability after tDCS. Considering the mechanism of an interhemispheric activity imbalance, bihemispheric tDCS (Bi-tDCS), which combines anodal tDCS to the M1 in the injured hemisphere with cathodal tDCS to the M1 in the non-injured hemisphere, has been investigated and reportedly improves interhemispheric imbalance (32–34). Recently, Bi-tDCS for patients with subacute stroke was reported to inhibit increased CST excitability and excessive inhibition from the non-injured hemisphere to the injured hemisphere during distal movements (34). These results suggest that Bi-tDCS, which can excite the CST, inhibits co-contraction and modulates the interhemispheric space. Thus, Bi-tDCS may aid recovery in patients with upper extremity paralysis. However, whether Bi-tDCS is more effective than anodal tDCS to the M1 remains unclear. Although several studies have focused on patients with chronic stroke, the effects of anodal tDCS and Bi-tDCS on distal muscle activity patterns and CST excitability during upper limb movements in patients with subacute stroke have not been comprehensively investigated. tDCS administered during the subacute period of recovery may yield more favorable rehabilitation outcomes. However, the extent of improvement may vary based on the upper limb motor dysfunction severity and recovery trajectory. We hypothesized that Bi-tDCS will decrease co-contraction and increase CST excitability during upper limb movements to a greater extent than anodal tDCS. Herein, we examined the effects of Bi-tDCS and anodal tDCS on distal upper limb motor function, co-contraction of forearm muscles during upper limb movements, and CST excitability in a patient with subacute stroke.

## 2. Materials and methods

### 2.1. Participant

**Figure 1:**
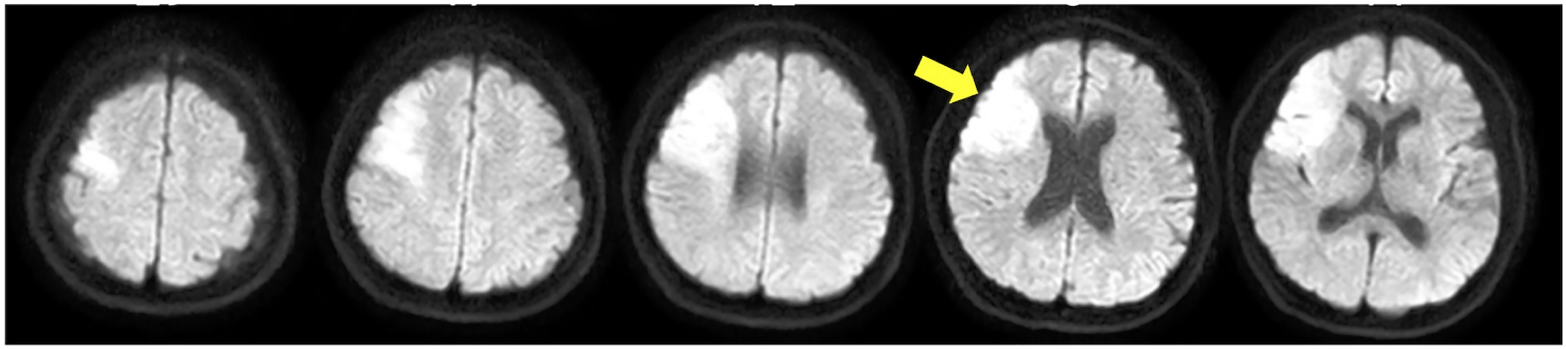
Magnetic resonance imaging scans showing the lesion sites in the patient.

This study included a 44-year-old woman with paralysis of the left upper and lower limbs due to a cardiogenic cerebral infarction. Nuclear magnetic resonance imaging showed a high-signal response in the right middle cerebral artery (Figure 1). The patient had an FMA score of 38 for the upper extremities 3 weeks after onset. The patient's shoulder joints received a perfect score; however, her hand joints and fingers were severely impaired, with scores of 2 and 0, respectively. The Box and Block Test (BBT) score was 0 for the left hand. In the Motor Activity Log, the scores were 0 for both “amount of use” and “quality of movement,” and she had difficulty in using the left upper limb in daily life. No apraxia, aphasia, or memory impairment was detected. The hospital's rehabilitation program included physical therapy, occupational therapy, and speech therapy. The time spent in rehabilitation was 1 h/day (seven times/week). The patient provided informed consent prior to the study onset. The study followed a retrospective research design. All procedures were approved by the ethics committee of Takarazuka Rehabilitation Hospital of Medical Corporation SHOWAKAI (ethics review number: 20211006; date of ethics approval: 12/14/2021) and were conducted in accordance with the principles embodied in the 1964 Declaration of Helsinki and its later amendments.

### 2.2. Experimental environment and protocol

The experimental conditions were (1) dorsiflexion of the wrist joint and (2) extension of the fingers in a simple random design. A monitor was set up in front of the participant (13.3 inches; height: 70 cm; distance from the participant: 40 cm). The participant placed her arms on the desk in a relaxed position and was able to move her wrists and fingers freely. We also asked that she watch the monitor during the task (Supplementary Figure S1). In both conditions, upward and downward arrows were used to indicate dorsiflexion and rest, respectively, for a total of five 4-s periods, based on the mark or symbol displayed on the monitor as well as beeps.

### 2.3. tDCS settings and protocols

**Figure 2:**
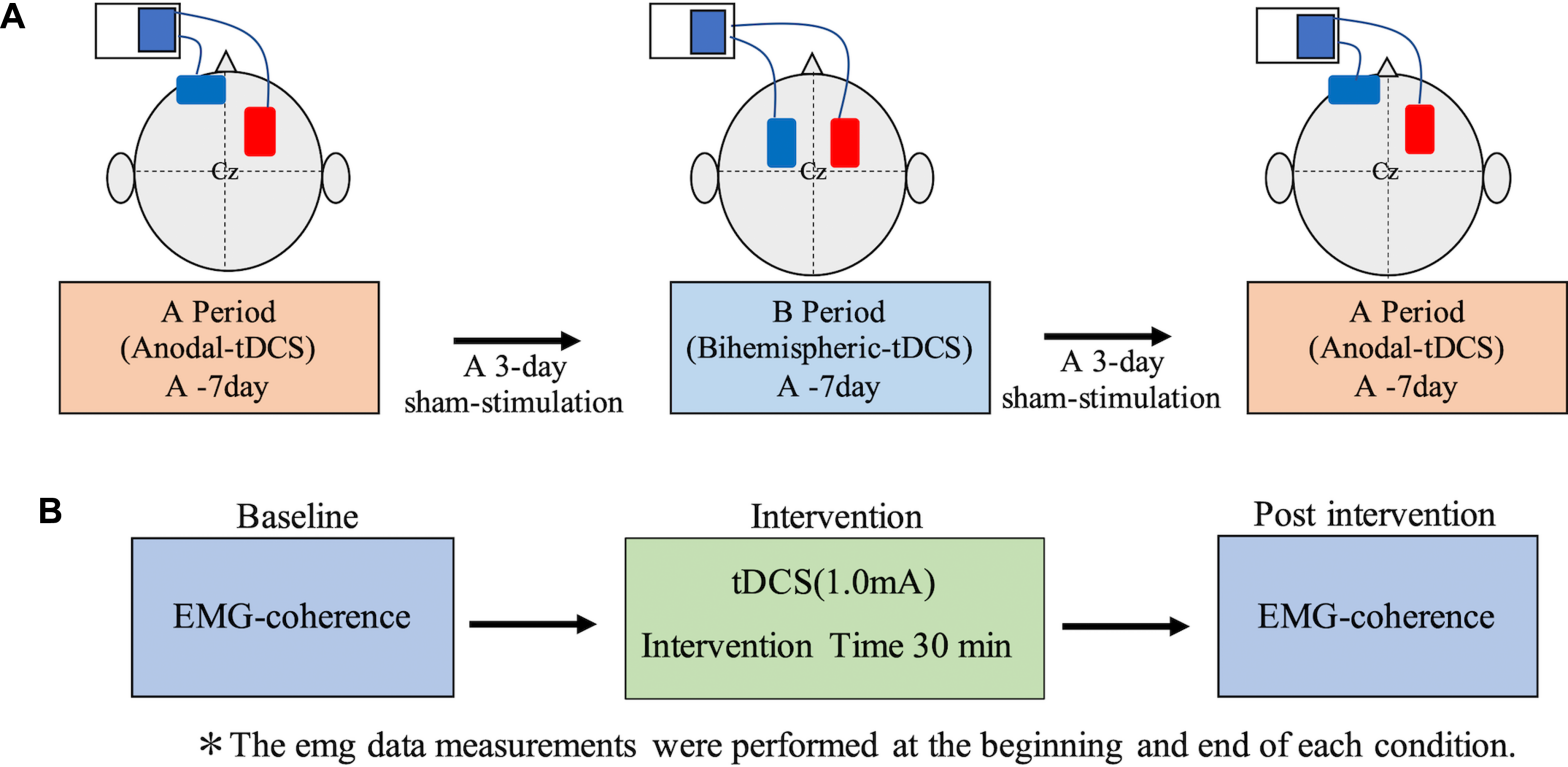
tDCS evaluation periods and electrode attachment locations. (**A**) In the anodal tDCS, the anode and cathode were placed on the injured and non-injured hemispheres, respectively. In the Bi-tDCS, the electrodes were positioned over the motor cortex bilaterally. The anode and cathode were placed on the injured and non-injured hemispheres, respectively. Each session lasted 1 week, with 3 days of sham stimulation in between. (**B**) Evaluation details before and after each session. Bi-tDCS, bihemispheric tDCS; tDCS, transcranial direct current stimulation.

The tDCS (DC-Stimulator Plus; Neuro Conn, Germany) was delivered via a stimulating electrode and two sponge pads (area: 35 cm^2^) with saline-soaked surfaces. A conductive gel was applied under the electrodes to reduce contact impedance. During transcranial DC electrical stimulation, the anode was placed on the M1 of the injured side, while the cathode was positioned on the contralateral forehead during anodal tDCS (A), in accordance with the International EEG 10–20 method. For Bi-tDCS (B), the anode was placed on the M1 of the injured side, whereas the cathode was placed on the M1 of the non-injured side (Figure 2); a constant current of 1.0 mA was applied for 30 min. The current density was 0.028 mA/m^2^, which is within the safety guidelines for tDCS (35, 36). The duration of stimuli at the beginning and end was 10 s. Occupational therapy with anodal tDCS or Bi-tDCS was carried out for 7 days each (total 21 sessions) in the following order: anodal tDCS, bilateral tDCS, and anodal tDCS. A 3-day sham stimulation period was used in the transition phase of each period to avoid any carryover effects between the periods. The sham stimulation was provided for the first 30 s to provide the participant with a sense of stimulation.

### 2.4. Assessment items

Motor function was assessed using the FMA, BBT, and surface electromyography (EMG). FMA is one of the most comprehensive scales for quantitatively assessing post-stroke motor disability and is recommended for stroke rehabilitation clinical trials. BBT is a functional outcome measure that characterizes the effectiveness of a specific treatment or rehabilitation plan.

### 2.5. Analysis methods and procedures

A wireless surface EMG (Gait Judge System; sampling rate: 1,000 Hz) was recorded at the paretic side of the proximal and distal portions of the extensor digitorum (ED) muscle and the proximal and distal portions of the flexor digitorum superficialis (FDS) muscle. Each skin site was shaved and cleaned with alcohol before electrode placement. To avoid the influence of electronic crosstalk, the distance between the electrodes was set to 20 mm. Raw EMG signals were bandpass-filtered using a zero-lag 4th-order Butterworth filter with cutoff frequencies of 5–450 Hz and were subsequently demeaned, rectified, and lowpass-filtered using a zero-lag 4th-order Butterworth filter with a 10 Hz cutoff frequency. The EMG signals were normalized by dividing them by the maximum amplitude obtained during upper limb movements. All preprocessing EMG procedures were performed following the Surface Electromyography for the Non-Invasive Assessment of Muscles guidelines (http://www.seniam.org).

EMG onset timing was defined as the point at which the EMG signal exceeded three standard deviations from the mean value of the EMG signal during the still states for the wrist and finger. We adapted linear interpolation over individual cycles of upper limb movements based on the timing of EMG onset to fit EMG signals to a normalized 100-point time base. The co-contraction index was calculated as the overlapping rate between the proximal ED and proximal FDS from normalized EMG signals.

EMG-EMG coherence analysis was performed on two time-series signals recorded from the proximal and distal ED muscle (agonist-agonist coherence) and from the proximal ED muscle and FDS muscle (agonist-antagonist coherence). Coherence was analyzed with the synchronization rate of two different time-series signals in each frequency band. There is a correlation between short-term motor unit synchronization and β-band coherence, indicating that these two phenomena, measured in the time and frequency domains, share the same mechanism. EMG-EMG coherence analysis was performed on full-wave rectified data; this method reportedly increase test-to-test reproducibility and reliability (37, 38). The analysis window consisted of 2,000 ms data segments extracted from each cycle after EMG onset. After selecting the EMG window, the data were passed through the Hamming window (window length: 2,000 ms; overlap: 1,000 ms) and subsequently concatenated. We defined the coherence between two concatenated EMG signals (x and y) as the square of the cross-spectrum normalized with the auto-spectrum according to the following formula:

(1) $$|R_{xy}(i){|}^{2}=\frac{{\left|\;f_{xy}(i)\right|}^{2}}{\;f_{xx}(i)f_{yy}(i)}$$

where *R_xy_* denotes the amplitude squared coherence for a given frequency (*i*), *f_xx_*(*i*) and *f_yy_*(*i*) indicate the x and y power spectra, respectively, and *R_xy_*(*i*) is the value of the cross-spectrum. Coherence can range from 0 to 1, with 1 representing a perfect linear correlation. Because the coherence of the beta band (15–30 Hz) was strongly reflected in CST activity, we calculated the beta band mean values for each cycle (37, 38). EMG data measurements were taken at the beginning and end of each condition, and the average of the five exercise tasks was analyzed. The BBT and FMA were calculated as the percentage change before and after training in each period, as follows:

(2) $$\mathrm{Variation}\;\mathrm{rate}=\frac{\mathrm{Post}-\mathrm{Pre}}{\mathrm{Pre}}*100$$

## 3. Results

### 3.1. Adverse events

The patient did not report any discomfort (convulsions, dizziness) or serious adverse events.

### 3.2. FMA and BBT progress

The progress of each clinical evaluation (A1/B1/A2) is described below. The BBT scores were 11/23/27, and the rate of change was 100%/109%/17.3%, indicating an increase in the score and improvement in the rate of change in the B stage. The FMA scores were 42/51/53, and the rate of change was 100%/17.3%/109%. When each item was subdivided, the wrist joints exhibited scores of 3/7/8, with a rate of change of 50%/133%/14.2%, and the fingers exhibited scores of 1/4/5, with a rate of change of 100%/300%/25%, indicating a predominant improvement in the B stage (Table 1). Both FMA and BBT scores exhibited superior improvement in the Bi-tDCS (B) period compared to the anodal tDCS (A) period.

**Table 1:** Clinical features of the case.

	Baseline	Anodal	Sham	Bihemispheric	Sham	Anodal	Sham
Fugl–Meyer Assessment-Upper extremity	38	42	42	51	51	53	53
Fugl–Meyer Assessment -Wrist	2	3	3	7	7	8	8
Fugl–Meyer Assessment -Finger	0	1	1	4	4	5	5
Box and Block Test	0	11	10	23	24	27	27

Both Fugl–Meyer Assessment and Box and Block Test showed superior improvement in the bihemispheric transcranial direct current stimulation (tDCS) period than in the anodal tDCS period.

### 3.3. EMG data analysis

Changes in EMG over time (baseline/A1/sham/B1/sham/A2) are depicted in Supplementary Figure S2. Muscle activity in the ED was 59.0%/40.8%/39.4%/36.6%/32.2%/28.8% for the wrist and 72.7%/50.1%/28.2%/59.5%/51.0%/39.5% for the fingers, indicating a decrease in muscle activity in the A period and an increase in the B period. The co-contraction index of the ED-FDS was 94.37%/92.61%/88.06%/48.75%/59.17%/38.77% for the wrist and 78.32%/74.3%/84.57%/61.36%/73.41%/61.28% for the fingers, indicating a decrease in the B period.

### 3.4. Intermuscular coherence results

**Figure 3:**
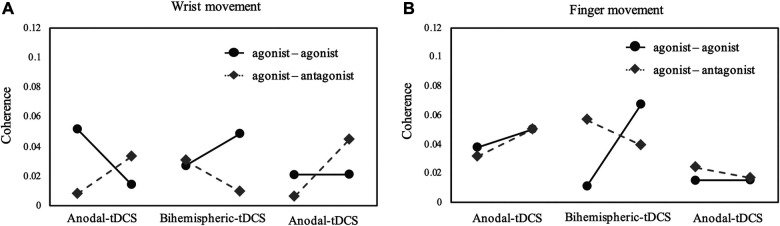
Coherence for each experimental week. (**A,B**) Coherence of beta-band activity in the ED-ED or ED-FDS. Period A and B were 1 week each, and sham stimulation was set at 3 days between each task. The results indicate that, compared with anodal tDCS, Bi-tDCS greatly improved the ED-ED coherence in the wrist and fingers. Bi-tDCS, bihemispheric tDCS; ED, extensor digitorum; FDS, flexor digitorum superficialis; tDCS, transcranial direct current stimulation.

Figure 3 depicts a longitudinal graph of intermuscular coherence results for the wrist and fingers. The results for agonist-agonist coherence during wrist movements were 0.051/0.014/0.026/0.048/0.021/0.020 at baseline/end of period A/end of sham stimulation/end of period B/end of sham stimulation/end of period A; on the other hand, the results for agonist-antagonist coherence during wrist movement were 0.008/0.033/0.009/0.006/0.044. Additionally, the results for agonist-agonist and agonist-antagonist coherence during finger movements were 0.037/0.050/0.010/0.067/0.015/0.015 and 0.031/0.049/0.056/0.039/0.023/0.016, respectively. An increase in agonist-agonist coherence and a decrease in agonist-antagonist coherence were observed in period B during wrist and finger movements.

## 4. Discussion

Herein, we longitudinally examined the effects of excitatory stimulation of motor-related areas on the injured side (anodal tDCS) and inhibitory stimulation of motor-related areas on the uninjured side (Bi-tDCS) on distal upper limb muscle activity patterns and CST excitability in a patient with subacute stroke. The results indicated that upper limb functional training with Bi-tDCS improved the muscle activity patterns and CST excitability during voluntary distal upper limb movements, compared with anodal tDCS. Motor function also showed a higher rate of change with Bi-tDCS than that with anodal tDCS. Interestingly, the coherence of anodal tDCS decreased the excitability and increased the index of co-contraction between the dynamic forearm muscles; contrastingly, the Bi-tDCS coherence increased the excitability and decreased the co-contraction index between the dynamic forearm muscles. These results indicate that tDCS can be used to stimulate the mechanism of neuroplasticity and more effectively treat patients with subacute stroke, depending on the method of use.

Applying anodal tDCS to the M1 increases MEPs as well as cortical-muscle and intermuscular coherence in the beta band (26, 27, 31). The similar pattern of change in MEPs and intermuscular coherence elicited by anodal tDCS may indicate a similar mechanism of action. In a study of cortical excitability induced by anodal tDCS and Bi-tDCS in the M1 regions, anodal tDCS increased excitability by 30%, whereas Bi-tDCS decreased it by 20% relative to the baseline MEP amplitude (39). In contrast, one study revealed similar changes in M1 excitability in both anodal tDCS and Bi-tDCS conditions (40). Although the effects of anodal stimulation on CST excitability between conditions may be comparable, CST excitability in the present study was improved with Bi-tDCS. This may be owing to the influence of interhemispheric inhibition (IHI). Previous studies reported that patients with severe motor paralysis use non-crossing descending fibers in the brain to control the paralyzed upper limb. Patients with severe upper limb motor paralysis are reportedly detrimentally affected when the M1 excitability on the contralesional side is suppressed, and the severity of the cortical motor system should be considered in the post-stroke cerebral cortex reorganization (41, 42). In this case, the patient had severe residual paralysis of the wrist and fingers; nonetheless, proximal paralysis was mild. Therefore, the combination with excitatory stimulation of the contralesional cortex was not considered effective. Post stroke, the contralateral hemisphere excitability increases due to a decrease in IHI from the injured to the contralateral side. This causes an interhemispheric imbalance between the bilateral cortices, which makes it difficult to increase the excitability of the diseased hemisphere (17–19). The patient infrequently used the paralyzed upper limb in daily life, and most daily activities were performed using the nonparalyzed upper limb. Based on these findings, we hypothesized that the patient had increased IHI from the unaffected hemisphere and unbalanced excitability in cortical motor areas. Therefore, we decided to perform Bi-tDCS, as anodal tDCS, whilst capable of increasing the CST excitability of the lesion, could not suppress the IHI from the contralateral lesion.

Dysfunction of the diseased CST increases the involvement of the reticulospinal tract, which is overactive to compensate for post-stroke limb movements. However, hyperexcitability of the reticulospinal tract is accompanied by spasticity, muscle hyperactivity, and abnormal muscle synergy in the upper extremities (43–45). In the anodal tDCS condition, the involvement of divergent descending mechanisms, such as the reticulospinal tract, and the residual interhemispheric imbalance would reduce CST excitability and worsen the co-contraction index and agonist-agonist coherence. However, in Bi-tDCS, excitatory stimulation to the lesion side and concurrent inhibitory stimulation to the contralateral side improved interhemispheric imbalance and increased CST excitability on the lesion side. This is supported by the results of agonist-agonist coherence. Based on these results, we hypothesize that a decrease in IHI increases CST excitability and decreases the co-contraction index. However, this is speculative because we did not measure the left-right difference index or IHI in this study.

This study has several limitations. First, the patient in this study had subacute stroke. Moreover, no control period was established. Therefore, although short-term changes resulted from tDCS, the possibility of spontaneous recovery must be considered (46, 47). Further, which patients would respond well to which tDCS protocols (stimulation site, intensity, stimulus density, and duration) remains undetermined (48). Furthermore, no standardized guidelines regarding the duration of the washout of the stimulation effect have been published (49). However, stimulation over consecutive days may cause cumulative effects with increased excitation effects. Although we used a 3-day washout period, at least 14-day interval between sessions would be optimal (50, 51).

In conclusion, this study showed improvements in upper extremity function, CST excitability, and muscle activity patterns following Bi-tDCS, as compared with anodal tDCS, in a patient with subacute stroke. These results suggest an improvement of the imbalance in interhemispheric activation. It is necessary to examine the effects of Bi-tDCS according to the stage from onset and severity of motor paralysis.

## Data Availability

The raw data supporting the conclusions of this article will be made available by the authors, without undue reservation.
