# Supplementary material for: Effect of bihemispheric transcranial direct current stimulation on distal upper limb function and corticospinal tract excitability in a patient with subacute stroke: a case study
Source: Front Rehabil Sci. 2023 Sep 5;4:1250579. doi: 10.3389/fresc.2023.1250579 (PMC10507690; doi:10.3389/fresc.2023.1250579)
Supplement: Supplementary file 1 [file Datasheet1.docx]

Supplementary Material

Effect of bihemispheric transcranial direct current stimulation on upper limb function and corticospinal tract excitability in a patient with subacute stroke: a case study

Takahiro Shiba^1*†^, Naomichi Mizuta^2,3†^, Naruhito Hasui^1,4^, Yohei Kominami^1^, Tomoki Nakatani^1^, Junji Taguchi^1^, Shu Morioka^3,4^

**†**These authors contributed equally to this work

*** Correspondence:** Takahiro Shiba
[hyougo.ot.shiba@gmail.com](mailto:hyougo.ot.shiba@gmail.com)

Shu Morioka
[s.morioka@kio.ac.jp](mailto:s.morioka@kio.ac.jp)

# Supplementary Figures


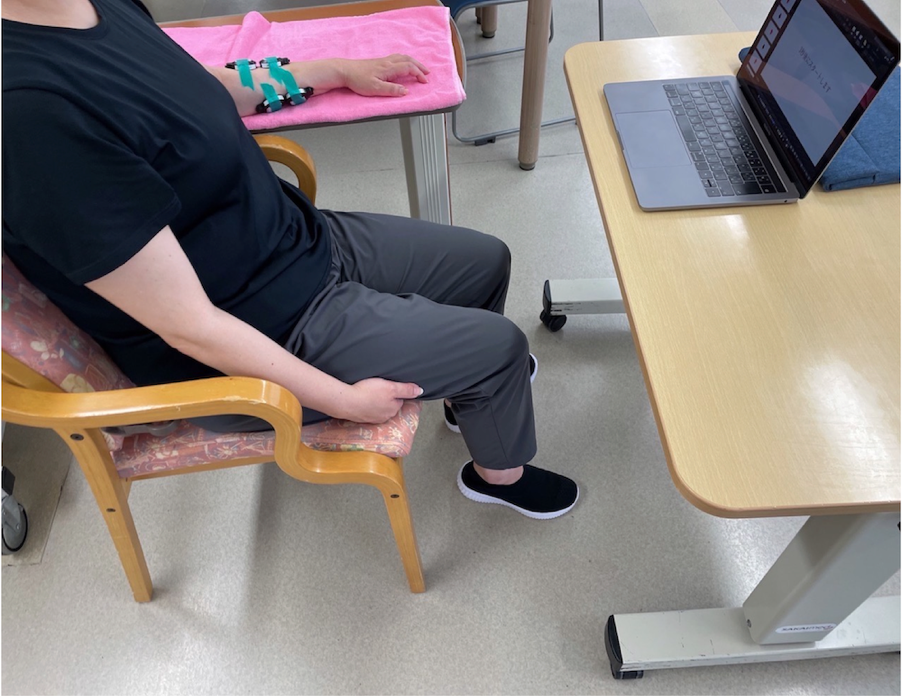


**Supplementary Figure 1.** Experimental setup in the upper limb task

The patient’s left side was the paralyzed side. To allow movements of the wrist and fingers in the direction specified by the sound stimulus, a towel and a desk were placed under the hand, and the upper limb was kept unloaded.

**
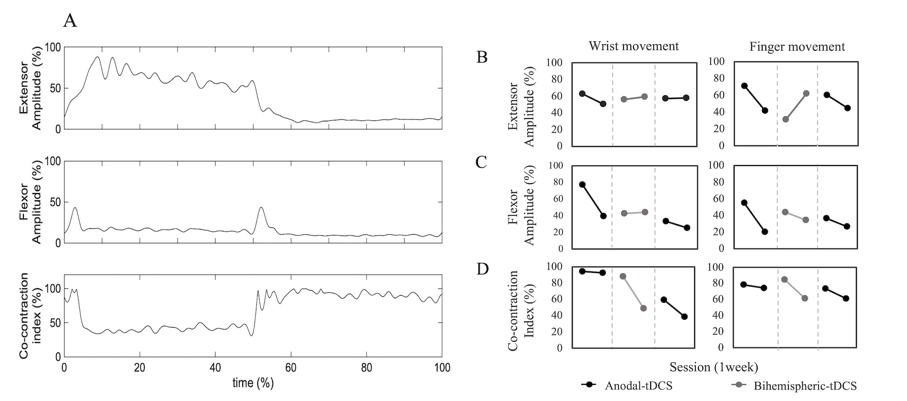
**

**Supplementary Figure 2.** Electromyography results for each experimental week

(A) Dorsiflexors, flexors, and co-contractors in order from top to bottom as an overview of the electromyogram data of the wrist joint during the task (one cycle). At the beginning of the exercise, the muscle activity of only the main driving muscle increased, and at the end of the exercise, the muscle activity of the antagonist muscle increased, with a decrease in the main driving muscle. (B–D) Electromyogram of the wrist and fingers. (B) Muscle activity of the extensor muscles during wrist joint and finger movements. (c) Muscle activity of the flexor muscles during the exercise. (D) Index of co-contraction of the extensor and flexor muscles.
